# A Transvenous Endovascular Approach in Straight Sinus Has Minor Impacts on Chordae Willisii

**DOI:** 10.3389/fneur.2022.725703

**Published:** 2022-02-11

**Authors:** Yuanliang Ye, Jiuyang Ding, Shoutang Liu, Tiancai Lan, Long Chen, Yingxue Wang, Bing Xia, Jianqing Yang

**Affiliations:** ^1^Department of Neurosurgery, Liuzhou People's Hospital, Liuzhou, China; ^2^School of Forensic Medicine, Guizhou Medical University, Guiyang, China; ^3^Department of General Surgery, Liuzhou People's Hospital, Liuzhou, China

**Keywords:** straight sinus, endoscopy, chordae willisii, anatomy, intervention

## Abstract

Cerebral dural sinuses contain different types of chordae willisii (CW). The transvenous endovascular approach, which has become an optimal method for the treatment of cerebrovascular diseases, such as malformation, fistula, and chronic intracranial hypertension, due to sinus thromboses, frequently uses retrograde navigation through dural sinuses. Whether or how much the endoscopic procedure damages the chordae willisii is often not well-assessed. In our study, an overall number of 38 cadaveric heads were analyzed for the distribution and features of the chordae willisii in the straight sinus. We used an endoscope on these samples mimicking a mechanical thrombectomy procedure performed in the straight sinus. Both endoscopic gross observation and light microscopic histological examination were used to assess the damages to the chordae willisii by the procedure. We found that the valve-like lamellae and longitudinal lamellae structures were mainly found in the posterior part of straight sinus whereas trabeculae were present in both anterior and posterior portions. We treated a group of samples with a stent and another with a balloon. The stent-treated group had a significantly higher rate of Grade 1 damage comparing with the balloon-treated group (*p* = 0.02). The incidence of damage to the surface of chordae willisii was also higher in the stent-treated group (*p* = 0.00). Neither the use of stent nor of balloon increased the rate of damage to chordae willisii during repeated experiments. These findings indicated that stent or balloon navigation through the straight sinus can cause minor damages to the chordae willisii and frequent uses of retrograde navigation through the straight sinus do not appear to increase the rates of damage to chordae willisii.

## Introduction

**Figure 1:**
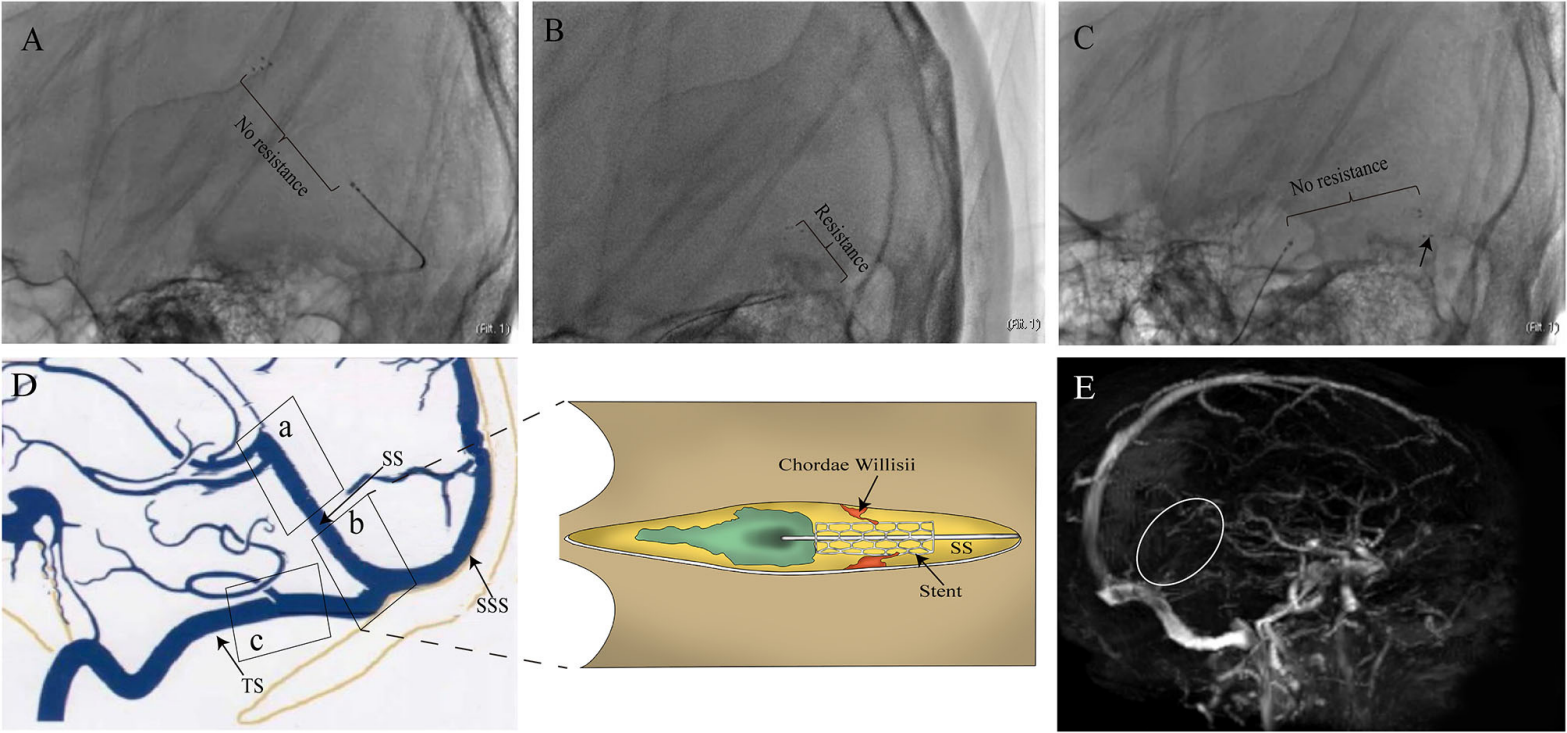
Images of the case that motivated this study. **(A)** 24-year-old woman with epilepsy and coma was admitted to the Department of Neurosurgery. The patient underwent cerebral angiography, which showed filling defects involving the SS and deep venous system. The patient then underwent mechanical thrombectomy with a stent. Resistance (from large to small suddenly) was encountered and led to dramatic movement of the patient's head during the withdrawal of the device in PoSS. The device was seemingly resisted by some anatomical structure within the sinus. No resistance was experienced when the stent moved through both AnSS and TS **(B,C)**. Although low-molecular-weight heparin and warfarin were used after surgery, the patient's consciousness returned to lethargy with recurrence of SS thrombosis (white circle) in 2 weeks after surgery **(E)**. The illustration of suspected causes of resistance in the SS **(D)**.

Most cerebral vascular diseases, such as vascular malformation (1, 2) and thrombosis (3, 4), involve the straight sinus (SS). The transvenous endovascular approach is considered a first-line surgical treatment for these diseases (4, 5). This intravascular intervention might result in iatrogenic damages to the chordae willisii (CW) that is present in the superior sagittal sinus (SSS). This damage may lead to subsequent exposure of collagen fibers resulting in thrombus formation (6). However, little literature has been found to support this hypothesis in the SS. During a transvenous endovascular thrombectomy procedure conducted at the Liuzhou People's Hospital of Guangxi Medical University, we encountered various amounts of resistance in the SS (Supplementary Video 1). Although administered with standardized anticoagulant therapy, the patient still experienced a recurrence of thrombosis 2 weeks after surgery (Figure 1). As it was confirmed that there was no venous stenosis in the SS, we suspected that the resistance we encountered could be caused by anatomical structures within the sinus. We speculated that the damage to internal structures in the SS might result in the exposure of collagen fibers, which activated a coagulation mechanism and contributed to thrombosis recurrence.

Radiology and standardized anatomical techniques have been employed to anatomically investigate the structure of the SS (7–9). For example, the composition of SS and the possibility of SS constituent layer dissection have been extensively described by using ultrastructural analysis (10–13). Endoscopic methods are also well-established and routinely utilized to examine the internal structures of the superior sagittal sinus (SSS) (14, 15). In this study, a high-definition rigid endoscopic procedure was carried out in the SS of cadavers by mimicking a mechanical thrombectomy (MMT) process. We observed CW in the SS and its morphological feature. The damage to CW *via* the endoscopic procedure was assessed through observations during the procedure and later by light microscopy.

## Materials and Methods

**Figure 2:**
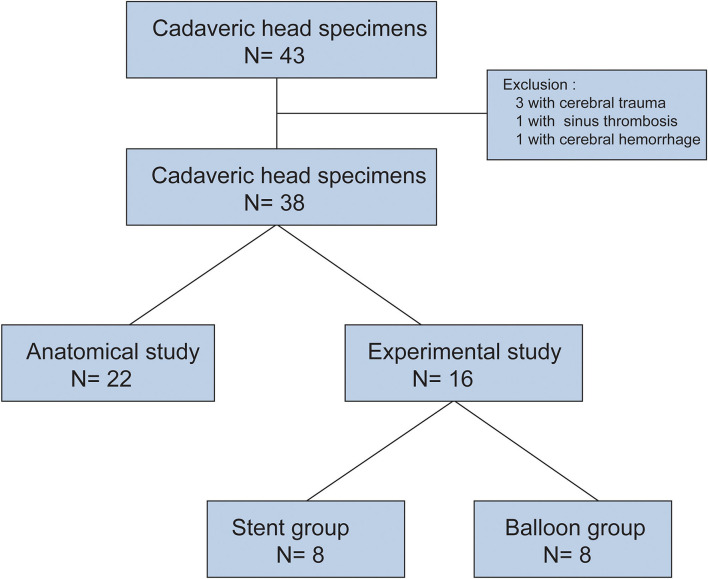
STROBE flowchart of cadaveric heads specimens.

A total of 38 cadaveric heads, 18 men and 20 women, were selected from autopsies that were taken at the Anatomy Department, Guangxi Medical University; 22 specimens were included for CW endoscopic assessment and 16 other cadaveric heads were included to mimic mechanical thrombectomy in the SS (Figure 2). Written consents of usage were obtained from donors or relatives of donors taking into consideration that they were all aged 18 years and above. The specimens belonged to 38 patients, 13 with a traumatic cause of death, 14 died due to pneumonia, 8 due to renal failure, and 3 due to hepatic failure. This research attained the approval of the ethics committee of Guangxi Medical University (IRB approval ID No. KY-E-01-01). Fixation of all cadaveric heads was performed using 10% formalin solution for a duration of 4–6 weeks. All cadavers with the following criteria were excluded: (1) craniocerebral trauma, (2) neurological diseases, and (3) diseases affecting the dural sinuses.

### Assessment of Morphological Characteristics of the Chordae Willisii by an Endoscope

Twenty-two cadavers were used in the endoscopic assessment of the CW in SS for the observational and descriptive study. The relevant clinical data was obtained from each individual's clinical record and the relatives. The data included gender, age, height, weight, comorbidities, and cause of death. The specimens' mean age was 65 ± 13.19 years (range 43–73), and they included 14 men and 8 women. Latex was not injected into veins or dural sinus. Scalp removal was performed with special attention to exposing the inion. A power tool was used to remove the calvaria. Removal of the occipital bone surrounding the inion was carried out using a rongeur until complete exposure of the confluence of sinuses and the bilateral transverse sinuses were achieved. After that, brain tissues surrounding the SS were resected en bloc, while the tissues surrounding the junction between SS and the great cerebral vein were subpially removed in a piecemeal fashion. A 4.5-gauge needle pointing toward the confluence of sinuses was inserted into the inferior sagittal sinus. Flushing of the SS and SSS was performed using tap water for removing blood clots. The cadavers were maintained in a prone position with the head fixed in a Mayfield head holder. A high-definition rigid endoscope (Karl Storz, German) with a 2.7 mm diameter and optics of 0° and 30° was inserted into the SS lumen and SSS from the confluence of sinuses. An endoscope equipped with a video system and a digital camera was used to document relevant findings.

### Mimicking Mechanical Thrombectomy in the SS

Sixteen cadaveric heads were used to evaluate the damage by MMT to CW in the SS. The specimens' mean age was 61 ± 12.48 years (ranging between 41 and 79), and they included 4 men and 12 women specimens. An Envoy guide catheter size 6-F (Cordis, Inc., Miami, FL, USA) was positioned at the transition from transverse to sigmoid sinuses through the posterior mastoid bone window at either side. In the group treated with a balloon, a microguidewire and a microcatheter were retrogradely introduced into the junction between the great cerebral vein and the SS. After confirming proper placement of the catheter by poster-anterior and lateral projection DSA radiography, the microguidewire's tip was kept at the SS's anterior third and the microcatheter was taken out, and a 5 mm rapid exchange balloon was placed instead of reaching to the anterior third of the SS along the microguidewire. In the stent group, a 4–20 mm Trevo Pro Vue stent retriever was delivered to the anterior third of the SS along the microcatheter. The dilated balloon or the stent was gently pulled back toward the sigmoid sinus. The same process was repeated 3 times.

### Assessment of Damage to the Chordae Willisii by an Endoscope

An endoscope was used to evaluate the damage to the CW; endoscope insertion was done into the SS lumen from sinuses' confluence. According to the degree of damage to the CW, a classification system was followed to express the grade of damage in this work, Grade 0: no damage; Grade 1: broken off at the surface of the CW; Grade 2: split away from the CW completely (Table 1).

**Table 1:** Simple damage classification system of CW during mimicking mechanical thrombectomy in the SS.

**Grade**	**Characteristics of damage on the CW**	
	**Endoscopic observation**	**Microscopic observation**
0	No damage	Complete endothelial lining and collagen fibers
1	Broken off at the surface of the CW	Damage on endothelial cell and collagen fibers partially ^*^
2	Split away from the CW completely	Damage both endothelial cell and collagen fibers completely

*CW, Chordae willisii*.

* *In the histological section of CW, the damage depth was less than half of the diameter; minor damage included grade 0 and grade 1*.

### Assessment of Damage to the Chordae Willisii by Light Microscopy

After tissue preparation, samples from the SS were subjected to microscopic examination. The H&E and Masson's trichrome (for collagen fiber) staining methods were utilized. Microscopic examination of the sections was performed by a Zeiss Axioskop Plus microscope (Carl Zeiss Microscopy) at x50, x100, and x400 magnifications. Images were acquired and stored by the Axio Vision software. The following classification system was used to record the microscopic damages: Grade 0: endothelial lining and collagen fibers remain intact; Grade 1: partial damage to the endothelial cell or collagen fibers; Grade 2: both endothelial cell and collagen fibers were completely damaged (Table 1).

### Statistics

Analysis of the statistical data was carried out using SPSS 22 for Windows (SPSS Inc., Chicago, Illinois). Categorical data, namely CW number, and the coverage percentage, were summarized using descriptive statistics. Summarizing the numerical data on the other hand was performed in form of means ± SDs. Independent-sample student's *t*-test was employed for comparing CW distribution in different segments of the SS. The non-parametric independent-sample test was used to compare the numbers of CW between SS and SSS. In addition, the non-parametric chi-square was used for comparing the CW damage between the balloon and stent groups. A *p*-value below 0.05 was considered of statistical significance.

## Results

### Types of Chordae Willisii in the SS

There were 210 CWs altogether in the 22 examined SS. The highest number in one SS was 13, and the lowest was 7. Details related to the number of CWs are shown in Table 2.

**Table 2:** Findings in 22 cadaveric heads specimens^*^.

**Specimen No**.	**Chordae willisiis in SS**			**Chordae willisiis in SSS**		
	**Valve-like lamellae**	**Trabeculae**	**Longitudinal lamellae**	**Valve-like lamellae**	**Trabeculae**	**Longitudinal lamellae**
1	6	3	1	8	2	2
2	6	4	1	7	5	4
3	3	4	2	9	5	2
4	4	3	3	5	8	4
5	2	4	2	10	7	5
6	3	2	3	6	4	5
7	4	4	2	9	5	3
8	5	2	2	8	8	5
9	3	4	2	10	4	3
10	4	2	1	6	8	3
11	6	4	3	6	7	6
12	4	3	1	5	7	2
13	6	4	1	8	7	2
14	3	3	1	7	4	3
15	5	3	2	6	5	3
16	6	4	2	10	3	6
17	4	5	1	7	5	8
18	6	3	2	8	6	3
19	3	6	1	6	3	5
20	6	3	2	6	4	5
21	4	2	1	9	4	7
22	2	3	3	4	5	3

* *Number of chordae willisii in straight sinus (SS) and superior sagittal sinus (SSS) by endoscopic assessment*.

**Figure 3:**
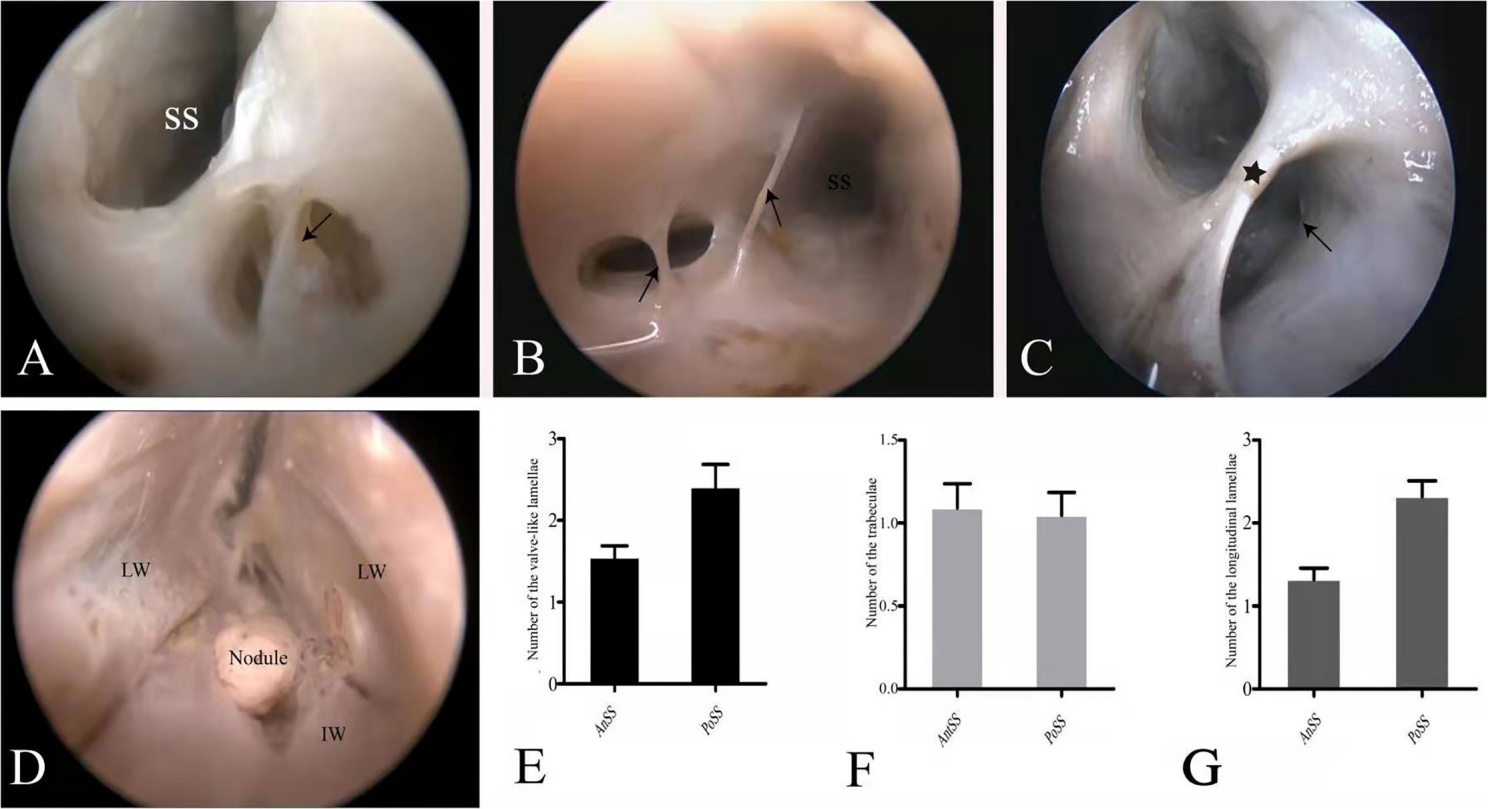
Endoscopic view of the chordae willisii (CW). A valve-like CW in the SS was located at the junction between the LW and IW (black arrowhead: valve-like chorda) **(A)**. Two trabeculae in the lumen of the SS (black arrowhead: trabeculae) **(B)**. A longitudinal chorda inside the sinus divided the lumen into two channels (black star: longitudinal chorda). A valve-like chorda is present in the sidewall of one channel (black arrowhead: valve-like chorda) **(C)**. Nodule present at the junction between the great cerebral vein and the SS **(D)**. Graphs show comparisons of the number of valve-like lamellae **(E)**, trabeculae **(F)**, and longitudinal lamellae **(G)** in the AnSS and PoSS.

The most commonly occurring CW was the valve-like lamellae that made up 45.23% of all CWs in the examined straight sinuses. The openings of the majority of valve-like lamellae were mainly located posteriorly in the SS and were found at the junction between the lateral and inferior walls (IW) of the sinuses (Figure 3A). The trabeculae, that constituted 36.19% of all CWs in our sample, was the second commonly occurring among forms of CW in the SS. They were found either at the center or the periphery of the lumen, appearing as either solitary or clustered structures (Figure 3B). The laminar chordae, which divided the lumen of the straight sinus into different diameters, were the least common form of CWs. Laminar chordae were observed either in the orifice or in the middle of the straight sinus and ran in horizontal, perpendicular, or oblique directions (Figure 3C). A nodule was found at the junction connecting the great cerebral vein and the SS (Figure 3D). The valve-like lamellae and longitudinal lamellae were mainly seen in the posterior portion of the straight sinus (PoSS) (Figures 3E,F). However, trabeculae were found in both the anterior and posterior portions of the straight sinus as well (Figure 3G).

### Comparison of the Number of Chordae Willisii to Superior Sagittal Sinus

The average numbers of CWs in each SS and SSS were 9.54 ± 1.68 and 16.90 ± 2.57, respectively. A significant difference was found in the number of CWs between SS and SSS (*p* = 0.00). There was no difference in the distribution of valve-like lamellae, trabeculae, and longitudinal lamellae between SS and SSS (*p* = 0.836).

### Effect of Stent/Balloon Damages to Chordae Willisii Observed by Endoscopy

**Figure 4:**
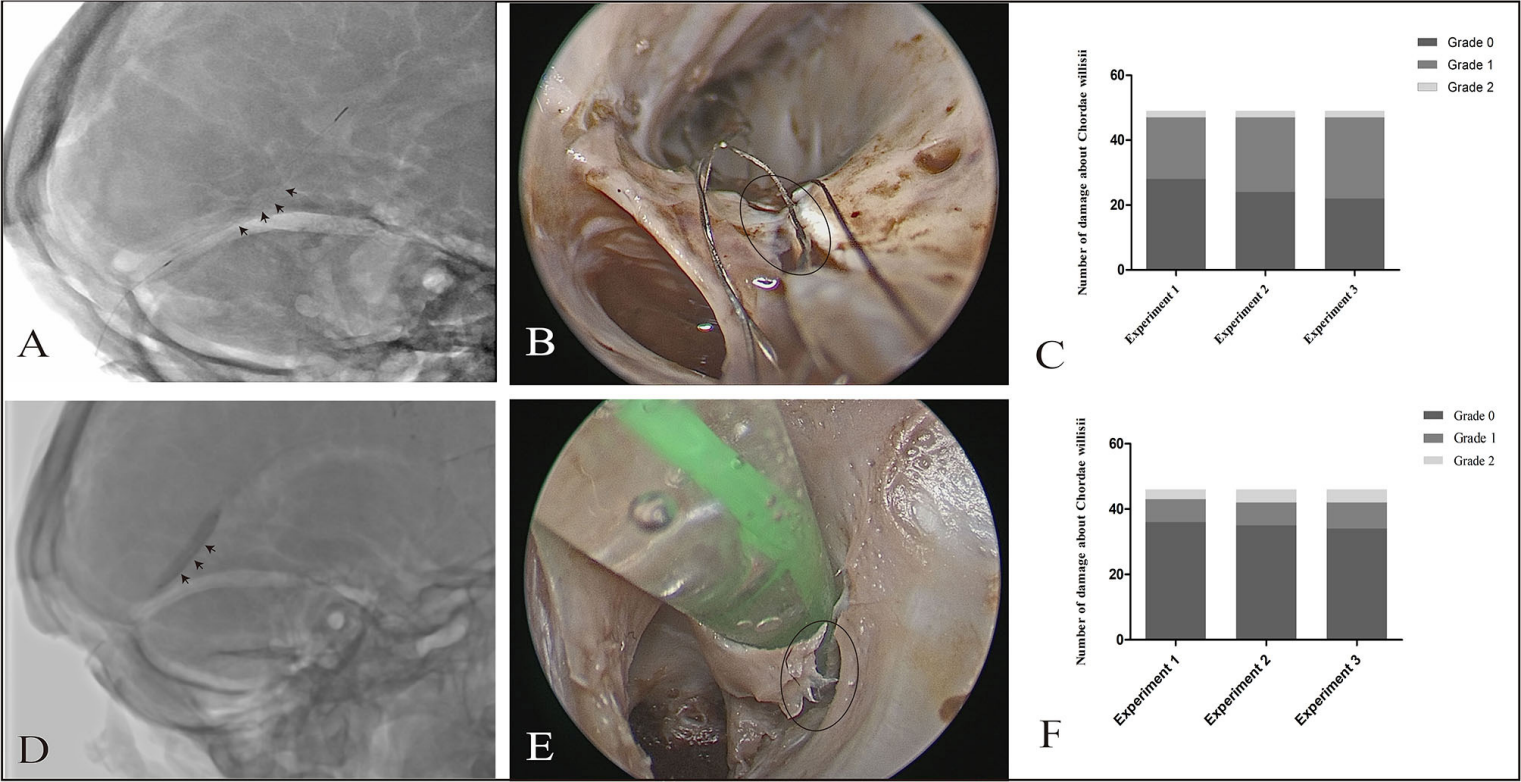
Effect of a stent/balloon on the CW in the SS. The stent was delivered to the anterior third of the SS along a microcatheter (black arrowhead: stent) **(A)**. Damage of Grade 1 **(B)** was observed by endoscopic view with 0° optics (black circle: damage area). Graphs show comparisons of the damage to the CW among three replicates in the stent group **(C)**. The balloon was delivered to the SS, dilated, and drawn slowly back to the sigmoid sinus (black arrowhead: balloon) **(D)**. Grade 2 damage was observed by endoscopy (black circle: damage area) **(E)**. Graphs show comparisons of the damage to the CW among three replicates in stent group **(C)** and balloon group **(F)**.

The damages to CW by either the stent or the balloon were recorded with a system that graded 0 as minimum damage and graded 2 as the maximum damage. In the stent group, number of cases with grades 0, 1, and 2 were 26 (53.1%), 21 (42.9%), and 2 (4.10%), respectively. Using a stent did not lead to a significant increase in damage to CW throughout the three repeated procedures (*P* = 0.511); In the balloon group, there were 34 (73.9%) cases showing Grade 0, 8 (17.4%) Grade 1, and 4 (8.7%) Grade 2. Using a balloon also yielded no significant increase in damage to CW throughout the repeated procedures (*P* = 0.88) (Figure 4).

There were no significant differences in the distribution of damage in both groups (*p* = 0.10). As per our observation, CW damage in the SS occurred at two sites, the surface and junction (between the inferior and lateral walls). The incidence of damage to the surface of CW was higher in the stent group than in the balloon group (*p* = 0.00).

### Effect of the Stent/Balloon Damages to Chordae Willisii as Microscopically Observed

**Figure 5:**
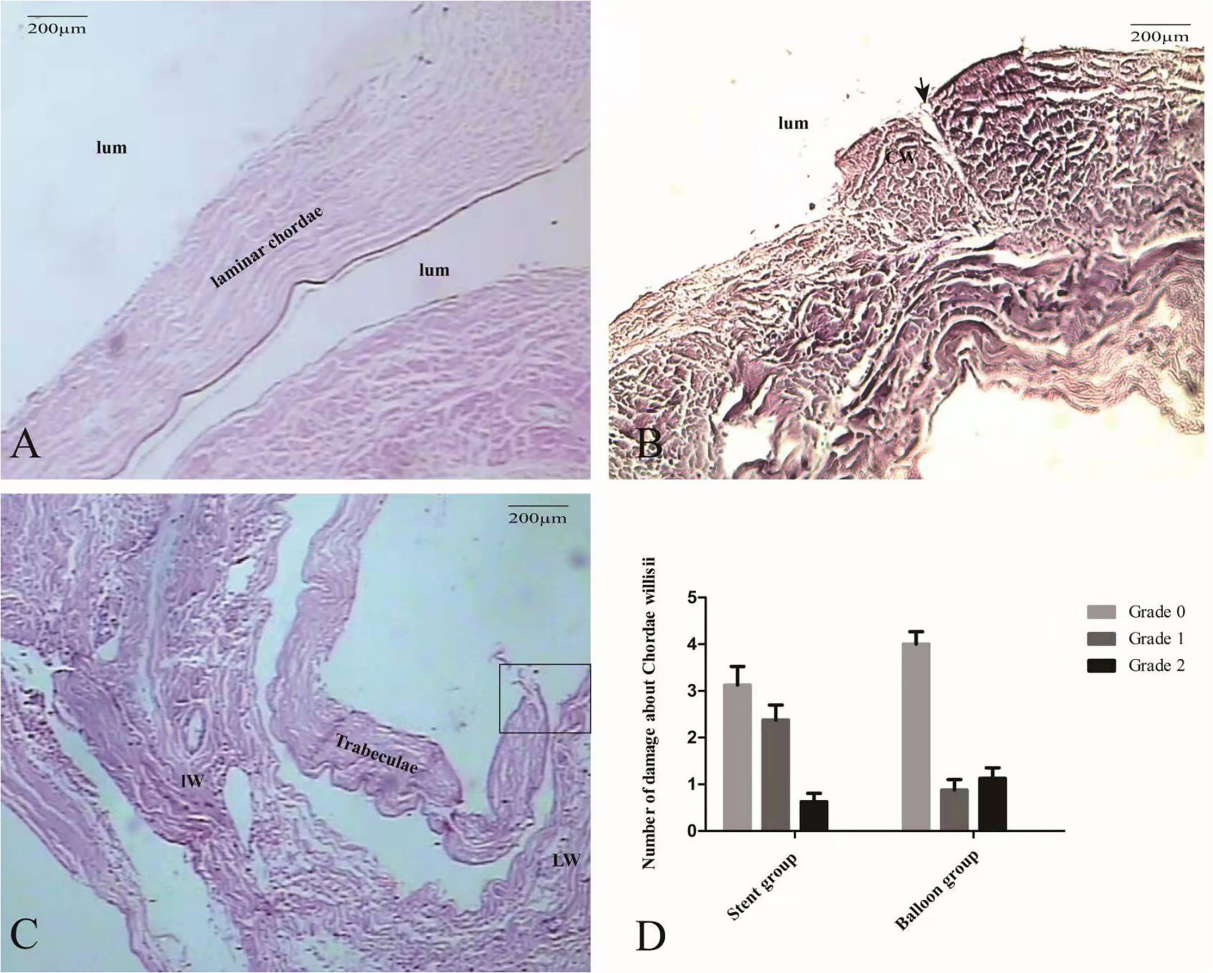
The morphological characteristics of intraoperative damage to the CW observed by microscopy. At Grade 0, the CW predominantly consists of collagen tissue and endothelial cells (HE, ×100) **(A)**. In a Grade 1 case, histopathological coronal sections showed the damage of endothelial cells (black arrow) and small area collagen fiber exposure **(B)**. In a Grade 2 case, a large area collagen fiber (square)was exposed completely (HE, ×100) **(C)**. Graphs show comparisons of the damage to the CW between the stent group and the balloon group **(D)**.

Histological staining of the Grade 0 cases revealed the presence of an endothelial lining with patent cell nuclei (Figure 5A). Subendothelial connective tissue containing collagen fibers and vessels was also observed. No smooth muscle fibers were observed in the CW. Histological staining also revealed a fissure at the surface of the CW in every Grade 1 cases (Figure 5B) and complete fragmentation of the CW in every Grade 2 cases (Figure 5C). Using stent led to significant increases in Grade 1 damage in the eight specimens than that observed in the balloon group (*p* = 0.02) (Figure 5D).

## Discussion

In this study, we observed the presence and morphological features of the CW in the SS using endoscopy and described the distribution of the CW. In addition, we assessed the degree of damages to the CW by either stent or balloon *via* endoscopic observation and histological investigation. We found that stent or balloon intervention could lead to mild damages to the CW, especially in the PoSS since more CWs in the PoSS. As far as we know, there is no data available in the literature providing a description of the impacts on the CW during transvenous endovascular treatment either *in vitro* or *in vivo*.

### Morphological Findings

Many neurosurgeons have recently turned their attention to the CW, which was recognized by early anatomists. Schmutz broadly identified the morphological characteristics of the CW in the SSS and divided the CW into three different forms, including valve-like lamellae, longitudinal lamellae, and trabeculae (16). The valve-like lamellae represented the most common form, whereas the longitudinal lamellae were the least common. Shao et al. and Sharifi et al. visualized and described the features of the CW structurally and topographically aided by a rigid endoscope. They also identified three types of CW in all examined specimens (14, 15). Similar to Schmutz, they also confirmed that CW was most commonly observed in the parieto–occipital region, and its most common type was valve-like. Additionally, Shao et al. also showed that cord-like structures are present inside the lumen of the cerebral vein ostia as well as networks of accessory cords in the venous lacunae. In our study, we also observed three types of CW in the SS and found that valve-like lamellae, which were located in the junction (between the inferior wall and the lateral wall), were the most common type of CW in the SS.

The previous investigation identified the presence of septa in the SS and found they were located in either the straight section of the SS or the whole SS (3). The differently shaped septa divided the SS into double or triple lumens (17–19). In our study, the size of the septum, which was identified as a longitudinal chordae, was broader than 2 mm. The longitudinal chordae were mostly detected at the anterior opening of the SS and extended into the confluence of sinuses.

### Histology Investigation of the Chordae Willisii

As a fetus develops, the intraluminal structures within the dural sinuses often originate from the cells of the primitive veins lying within the epidural space, where these cells start merging and forming different-sized structures into venous sinuses. Throughout SS formation, dural folds form as the brain tissue is compressed between the mesencephalon and rhombencephalon (20).

Accurate knowledge about the histological structure of the SS should be particularly beneficial to interventional neuroradiologists performing procedures inside the dural sinuses as this knowledge may significantly affect the difficulty and risk of the procedure. In the SS, the thicker inferior wall in comparison with the lateral ones was because it has a larger number of tissues (e.g., connective tissue, blood vessels, elastic fibers, and nerve fibers). The collagen fibers' percentage was less in the PoSS, in comparison with the SSS or the TS. In addition, transmission electron microscopy showed that muscle fibers are present in the inferior wall of the SS, extending between its junction with the great cerebral vein and the confluence of sinuses (11). The CW in the posterior cranial fossa consists predominantly of collagen tissues and seldom contains endothelial-lined vascular channels. The CW showed a smooth surface with a single layer of endothelial cells coverage. The occurrence and thickness of the CW vary among different dura sinuses (13). In our study, we found larger amounts of collagen fibers both in longitudinal lamellae and trabeculae. Blood vessels were seen in longitudinal lamellae.

### Clinical Significance on MMT Studies

With endovascular techniques, interventional neuroradiology must frequently utilize retrograde navigation through the main dural sinuses to treat arteriovenous malformations or fistulas, and sometimes idiopathic chronic intracranial hypertension resulted from sinus thromboses or hypertrophic arachnoid granulations. This study revealed that, when similar procedures are used frequently, iatrogenic damage to the lamellae and trabeculae can minimally occur upon exposure of collagen fibers in small areas. Clinically, intraoperative applications of anticoagulants decrease the incidence of thrombosis. However, in rare cases, the balloon or the stent may cause severe damages to the CW, and therefore, increase the area of exposure of collagen fibers, resulting in possible activation of blood coagulation. The presence of valve-like lamellae and longitudinal lamellae in the SS may render the catheter moving into a blind cavity in the PoSS. A catheter may move into the narrow channels surrounded by trabeculae and the sinus wall causing the unsuccessful opening of the stent. Thus, detailed knowledge of the SS anatomy may be helpful in avoiding procedural difficulties and achieving higher success rates.

### Limitations

The current study was limited by some factors. First, it did not guide how to avoid intraoperative damage to the intraluminal structures throughout transvenous endovascular therapy. However, knowledge of the internal structures of the SS, especially the distribution of the CW in different segments of the SS, provides a foundation for surgeons to navigate through technical problems. Second, it is uncertain which type of damage to the CW has a greater hemodynamic impact, but it is very likely that the seriousness of the damages affects the risk of thrombosis.

## Conclusion

With the aid of endoscopy, we described the distribution of intraluminal structures in the SS. We also demonstrated the minor damages to the CW caused by a stent or a balloon when they were navigated through the SS. Moreover, frequent usage of the retrograde navigation through the SS did not appear to increase the rate of damage to the CW.

## Data Availability Statement

The raw data supporting the conclusions of this article will be made available by the authors, without undue reservation.

## Ethics Statement

The studies involving human participants were reviewed and approved by Guangxi Medical University (IRB approval ID No. KY-E-01-01). The patients/participants provided their written informed consent to participate in this study.

## Author Contributions

JY, YY, and BX contributed to the thought of concept and design. YY and JD are responsible for the acquisition of data, illustration, and drafted the manuscript. YY, JD, SL, TL, LC, and YW analyzed and interpreted the data. YY and BX are responsible for the statistical analysis. The supervision of the study was conducted by JY. All authors contributed to the article and approved the submitted version.

## Funding

This work was supported by the Clinical Research of Guangxi Autonomous Region (Grant No. Z 20200158) and the Clinical Research of Liuzhou General Hospital (Grant No. LRY202015) to YY, Guizhou Scientific and Technological Plan Project [Grant No. ZK (2021) regular 487] to BX, and Research Foundation for Advanced Talents of Guizhou Medical University [Grant No. University Contract of Doctors J (2021) 014] to JD.

## Conflict of Interest

The authors declare that the research was conducted in the absence of any commercial or financial relationships that could be construed as a potential conflict of interest.

## Publisher's Note

All claims expressed in this article are solely those of the authors and do not necessarily represent those of their affiliated organizations, or those of the publisher, the editors and the reviewers. Any product that may be evaluated in this article, or claim that may be made by its manufacturer, is not guaranteed or endorsed by the publisher.
